# Analyte-mediated growth of gold nanoparticles for non-aggregation-based colorimetric detection of manganese(ii)

**DOI:** 10.1039/d6ra02740g

**Published:** 2026-06-02

**Authors:** Melisew Tadele Alula, Mildred Lesang Madingwane

**Affiliations:** a Department of Chemical and Forensic Sciences, School of Pure and Applied Sciences, Botswana International University of Science and Technology Plot 10071, Private Bag 16 Palapye Botswana alulam@biust.ac.bw +267-4900102 +267-76126741

## Abstract

Intensive industrial applications of manganese have increased its production from mineral sources, leading to increased emissions into surface and groundwater. The availability of manganese in water sources may result in debilitating health effects. It is, therefore, important to design an analytical method to monitor manganese in water sources. In this study, the localized surface plasmon resonance (LSPR) property of gold nanoparticles (AuNPs) is considered for sensing Mn^2+^. The extinction intensity of AuNPs increases on the addition of Mn^2+^. The extinction intensity of AuNPs increased linearly with the concentration of Mn^2+^ in the range of 0.5–25 µM. The limit of detection (LOD) in the linear range was computed to be 0.269 µM. Mn^2+^, producing the highest extinction response among the tested cations, shows the excellent selectivity of our method towards Mn^2+^. The applicability of the method in real sample analyses was tested using borehole groundwater. The recovery rates from the groundwater analyses ranged from 101–108%, showing the high accuracy of the method in the determination of Mn^2+^. The results show the potential of the method in the determination of Mn^2+^ in various environmental samples. In conclusion, this work shows the potential of the analyte-mediated colorimetric method for the detection of various target analytes.

## Abstract 2

Colorimetric method for detection of Mn^2+^ is developed. Localized surface plasmon resonance (LSPR) property of gold nanoparticles (AuNPs) is considered for sensing Mn^2+^. The extinction intensity of AuNPs increases when they interact with Mn^2+^.

## Graphical abstract

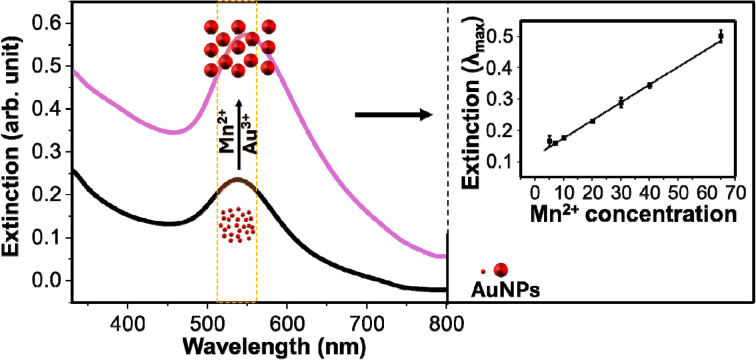

## 1. Introduction

Manganese is among the essential elements involved in the formation of enzymes such as arginase, glutamine synthetase, and superoxide dismutase.^1^ Besides its biological roles, the attractive properties of manganese have increased its metallurgical and industrial applications. For example, manganese oxide is used in the production of materials for different applications, including dry cell batteries, fireworks, glazes, livestock supplements, catalysts, fertilizers, ceramics, and fungicides.^2^ Because of its extensive applications, a large amount of manganese is produced and has contributed to the development of the economy of developing and developed countries.^3^ The high demand for manganese has increased the release of manganese to surface and groundwater through various activities, consequently resulting in adverse effects on human health.^4^ Drinking water and ingestion through food are the main routes by which humans are exposed to manganese. Despite its roles in enzyme formation, higher levels of manganese have detrimental health effects that require attention.^5^ Therefore, regular monitoring of manganese in water is highly important.

Inductively coupled plasma-mass spectrometry, atomic absorption spectroscopy, inductively coupled plasma-optical emission spectrometry, and X-ray fluorescence spectrometry are some of the techniques used in the detection of manganese.^6–9^ Although these techniques provide high sensitivity, their high cost, time-consuming procedures, and requirement of expensive instruments and skilled personnel limit their applications in routine analyses. Recently, detection systems using nanomaterials with fluorescence, surface plasmon resonance, LSPR, surface-enhanced Raman spectroscopy (SERS), and surface-enhanced infrared absorption (SEIRA) have been used for the detection of different analytes of interest.^10,11^ AuNPs and silver nanoparticles (AgNPs) are among the nanomaterials that have been used extensively in the determination of various analytes through colorimetric sensing.^12,13^ In the visible spectrum, AuNPs have distinctive LSPR characteristics that cause strong optical absorption and scattering. The optical properties of the nanoparticles change as the particle features and the dielectric environment change due to molecules that can form interactions with the nanoparticles. The change in optical properties can be associated with the identity and concentration of the target analyte.^14^

The colorimetric method based on LSPR involves monitoring the LSPR band shifts or intensity. The band shifts and intensity changes are initiated through two mechanisms, namely, aggregation/dispersion and non-aggregation. The aggregation/dispersion approaches are the most extensively used strategies and involve measuring the change (shift/damping) in the bands due to aggregated and dispersed nanoparticles.^15^ Even though colorimetric methods based on aggregation have been used extensively and are relatively sensitive, they have two basic limitations. Self-aggregation of the colloidal nanoparticles is the first problem. Different factors in the detection system, such as pH, temperature, salt, and charged molecules, can induce aggregation of the nanoparticles in the absence of the target analyte, which leads to a false positive outcome. Complex and time-consuming modification of the nanoparticles with analyte-recognition reagents and suitable functional organic ligands are additional problems.^15^

In recent years, non-aggregation-based sensing systems that involve particle growth or etching of the particles have become an alternative approach. The etching and growth result in the evolution of the size, shape and dielectric environment of the nanoparticles. The evolution of new particle features changes due to the presence of the target analyte, resulting in a plasmon band shift.^10,15,16^ For example, Yang *et al.*, (2014) and He *et al.*, (2025) reported etching of triangular silver nanoprisms and gold nanorods for the detection of DNA and deoxynucleotidyl transferase, respectively.^17,18^ Similarly, the size of the preformed plasmonic nanoparticles can further grow in the presence of the target analyte. This growth may proceed as a function of a certain range of concentrations of the target analyte. For example, Jafarinejad *et al.*, (2017) developed a colorimetric method for the simultaneous sensing of catecholamine neurotransmitters. The color of the reaction mixture containing gold nanorods and silver nitrate changes upon the addition of the neurotransmitters, as silver nanoparticles are deposited on the gold nanorods.^19^ Detection of formaldehyde has been reported by growing AgNPs on silver nanoclusters templated by polymethacrylic acid.^20^ Unlike the aggregation/dispersion strategy, the non-aggregate approach avoids a false positive signal due to the auto-aggregation of the nanoparticles.^21^ The nanoparticles in these types of detection systems are normally label-free. Therefore, labelling procedures, which are time-consuming and complex, can be avoided.^22^ Moreover, unlike the aggregation approach, the non-aggregation based process exhibits enhanced selectivity.^23^ Despite the non-aggregated approach having these advantages, non-aggregation based colorimetric assays for Mn^2+^ ions are limited.^23^

In this study, we develop a colorimetric method for the determination of Mn^2+^ ions using AuNPs. The approach is based on the growth of small-sized AuNPs in the presence of Au^3+^ and Mn^2+^. Addition of Au^3+^ and Mn^2+^ to the AuNP colloids changes the plasmonic properties of the AuNPs. This change demonstrates Mn^2+^-mediated growth of the AuNPs. The wine-red colored AuNPs change to pink, and this is accompanied by enlarged particle sizes. The intensity of the plasmonic band increased with Mn^2+^ with a slight band shift. The increment is a function of the concentration of Mn^2+^ within a certain concentration range. Based on this, the determination of Mn^2+^ in borehole groundwater is realised. This method shows excellent selectivity. To the best of our knowledge, this is the first report in which Mn^2+^-mediated growth of AuNPs is observed and used for its detection.

## 2. Experimental section

### 2.1. Chemicals

Analytical grade chemicals were used as received from the suppliers with no further treatment. Aqueous solutions were prepared using Millipore double-deionized water. Gold(iii) chloride trihydrate (HAuCl_4_·3H_2_O, 99.995%), silver nitrate (AgNO_3_), glacial acetic acid (CH_3_COOH, 99.8%), anhydrous sodium acetate (CH_3_COONa, 99%), iron(iii) chloride hexahydrate (FeCl_3_·6H_2_O, 98%), mercury(ii) nitrate (Hg(NO_3_)_2_, 98%), cobalt(ii) nitrate hexahydrate (Co (NO_3_)_2_·6H_2_O, ≥98%), manganese(ii) sulfate monohydrate (MnSO_4_·H_2_O), and lead(ii) nitrate (Pb (NO_3_)_2_, ≥99%) were purchased from Sigma-Aldrich. Iron(ii) sulphate heptahydrate (FeSO_4_·7H_2_O, 98+%), zinc sulphate heptahydrate (ZnSO_4_·7H_2_O), sodium chloride, and magnesium nitrate hexahydrate (Mg(NO_3_)_2_·6H_2_O) were obtained from Rochelle chemicals. Trisodium citrate dihydrate (Na_3_C_6_H_5_O_7_·2H_2_O, 99%) was purchased from Univar. Zinc chloride (ZnCl_2_, 98%) and calcium nitrate tetrahydrate (Ca(NO_3_)_2_·4H_2_O) were obtained from Merck. Potassium nitrate (KNO_3_) was purchased from Minema. Barium chloride dihydrate (BaCl_2_·2H_2_O) was obtained from Associated Chemical Enterprises. Nickel sulphate hexahydrate (NiSO_4_·6H_2_O) was purchased from Alpha Chemicals.

### 2.2. Preparation of AuNPs

A previously reported method with minor modification was employed to prepare the AuNPs.^24^ First, 100 mL of 0.25 mM HAuCl_4_ aqueous solution was transferred to a conical flask and stirred on a hot plate to boil. To the boiled solution, 1 mL of 5% trisodium citrate solution was added rapidly. The reaction mixture was allowed to boil. After 20 min, the colour of the solution appeared wine-red and was removed from the hot plate and placed in an ice bath to quench the reaction. The resulting colloidal AuNPs were stored in the refrigerator and used for this study.

### 2.3. Instrumentation

The UV/visible absorption spectra were collected using a Thermo-Fischer spectrophotometer using a 1 cm path length quartz cuvette as the sample cell. A Talos F200X G2 transmission electron microscope was used to collect the TEM images.

### 2.4. Detection of manganese(ii)

**Scheme 1:**
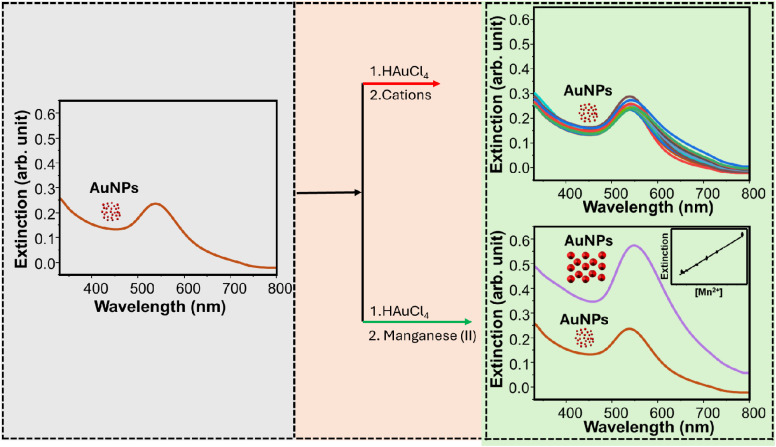
Strategy used for developing the sensing system.

Manganese(ii) was detected using AuNPs as plasmonic nanomaterials. In a typical experiment, 400 µL of the synthesized AuNPs, 750 µL of 0.3 mM HAuCl_4_, 750 µL of different concentrations of Mn^2+^, and 100 µL of acetate buffer (pH 3.6) were transferred to a 2 mL Eppendorf tube and kept for 30 min, and then UV/visible spectroscopy measurements were carried out. The scheme for the detection system is demonstrated in Scheme 1.

### 2.5. Determination of Mn^2+^ in borehole groundwater

The reaction conditions used in the detection of Mn^2+^ in deionized water were used to determine Mn^2+^ in borehole groundwater samples. The borehole groundwater was filtered with a filter membrane (0.22 µm) and diluted 10 times. Mn^2+^ solution was spiked to obtain the desired concentrations.

## 3. Results and discussion

### 3.1. Properties and characterization of the AuNPs

**Fig. 1:**
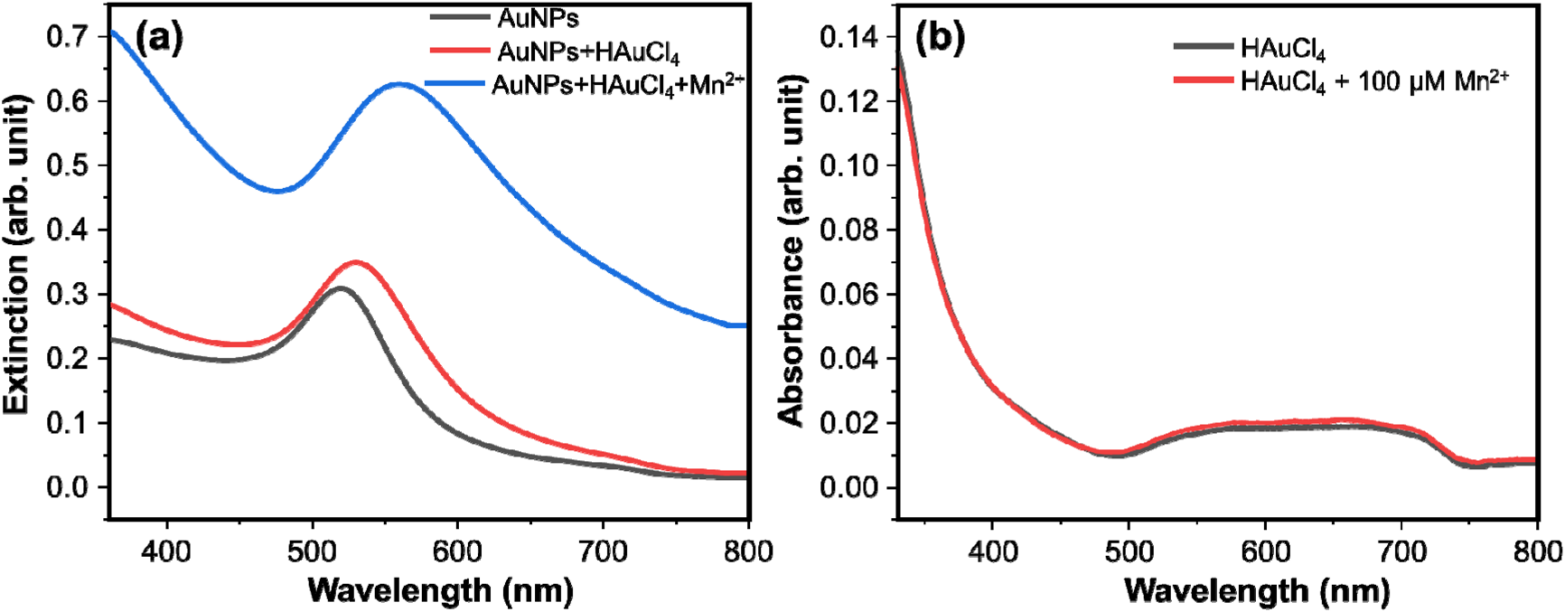
(a) Extinction spectra of AuNPs: as-prepared AuNPs, AuNPs + HAuCl_4_, and AuNPs + HAuCl_4_ + Mn^2+^. (b) UV/visible absorption spectra of HAuCl_4_ and HAuCl_4_ + 100 µM Mn^2+^.

A simple heat-induced wet chemical reduction method was used for the synthesis of the AuNPs. Trisodium citrate served as a reducing and capping agent.^25^ Spherical AuNPs with a plasmonic band centred at around 520 nm were formed. We strategize a detection system based on the growth of pre-synthesized AuNPs using Au^3+^ as a precursor for the formation of Au^0^ and a growth-mediated analyte. The plasmonic properties of solutions containing AuNPs alone, AuNPs + Au^3+^, and AuNPs + Au^3+^ + Mn^2+^ were investigated. As shown in Fig. 1a, the plasmonic band for the AuNPs (*ca.* 520 nm) red-shifted for the AuNPs + Au^3+^, accompanied by a slight increment in the extinction intensity, showing a slight increment in the size of the AuNPs (red curve). This is ascribed to the reduction of Au^3+^ catalysed by AuNPs and the mild reducing property of citrate at room temperature.^26–28^ The increment in intensity of the plasmonic extinction band is due to the larger size of the spherical AuNPs that arises because of the increment in the extinction coefficient.^15^ The red-shifted LSPR band is pronounced for the AuNPs + Au^3+^ + Mn^2+^ system, showing the formation of more AuNPs that are deposited on the pre-synthesized AuNPs (blue curve). Furthermore, we investigated whether the pre-synthesized AuNPs had a role in assisting the reduction of Au^3+^ to Au^0^. In the absence of AuNPs, when only Au^3+^ and Mn^2+^ were incubated for 60 min, no characteristic plasmonic spectrum for AuNPs was observed (Fig. 1b), showing that there is no reduction of Au^3+^ to Au^0^ in the absence of AuNPs as seeds. Because the AuNPs were prepared using trisodium citrate as a reducing agent, the role of trisodium citrate in the absence of these seeds was investigated. Two reaction mixtures lacking AuNP seeds were prepared: (i) trisodium citrate and HAuCl_4_ and (ii) trisodium citrate, HAuCl_4_, and Mn^2+^. UV/visible spectroscopy of the reaction mixtures confirmed that no new AuNPs were formed (Fig. S1). This implies that the trisodium citrate used in the synthesis of AuNP seeds is not independently involved in particle growth and hence does not reduce Au^3+^ under room temperature reaction conditions. However, the band around 364 nm in the presence of Mn^2+^ shows the interaction of Mn^2+^ and citrate. Therefore, the deposition and reduction of AuCl_4_^−^ occurs in the presence of AuNPs. Conclusively, the reduction of Au^3+^ requires AuNPs seeds to surve as nucleation centers for the growth of larger nanoparticles.^27,28^ The increment of the spectral intensities with time supports the growth of the nanoparticles (Fig. S2).

**Fig. 2:**
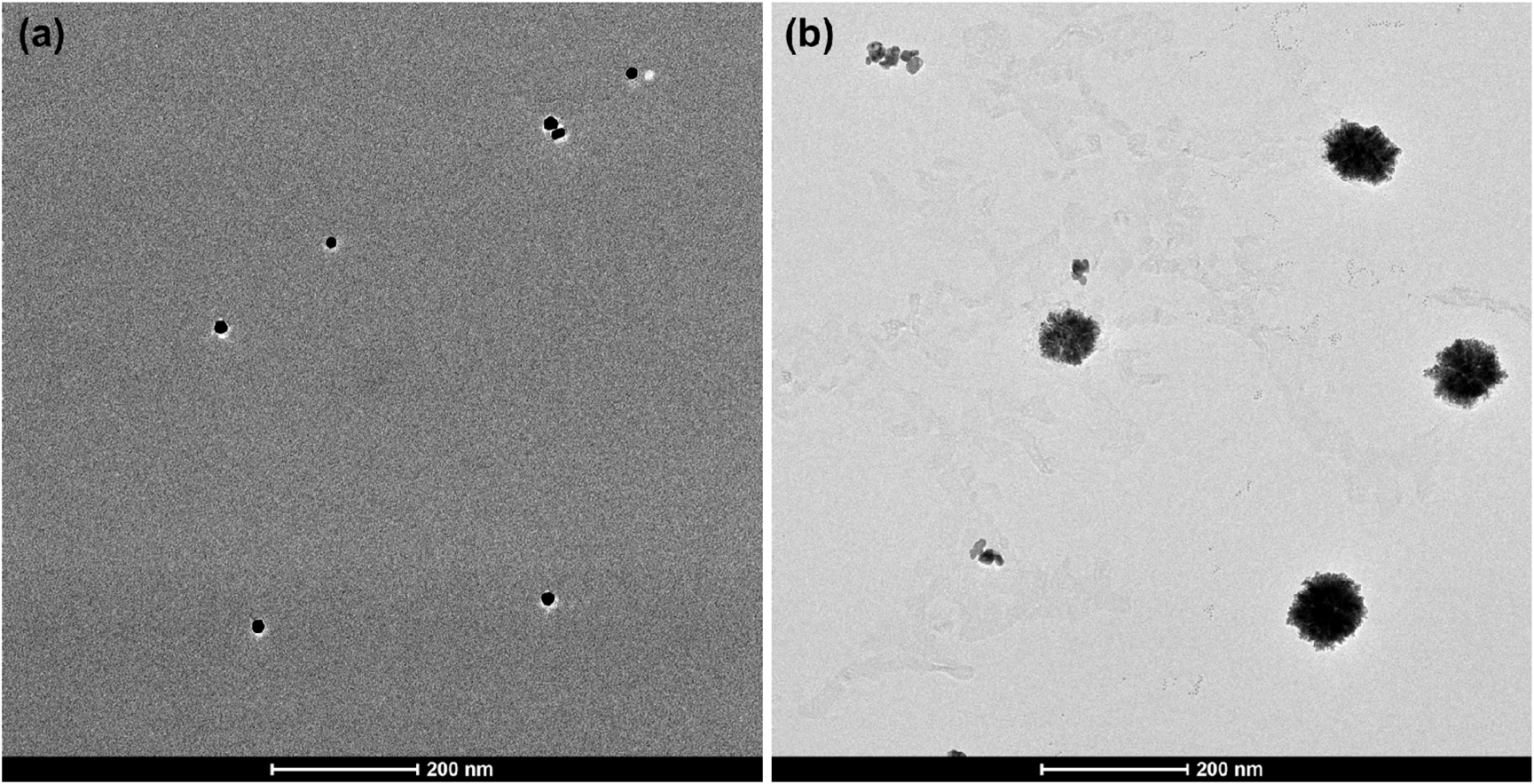
TEM images of (a) AuNPs and (b) AuNPs after the addition of Mn^2+^.

The response of manganese to AuNPs was studied further using TEM. In the absence of Mn^2+^, the LSPR band of the AuNPs is narrow and centred around 520 nm (Fig. 1a, black curve). Correspondingly, the TEM image shows uniformly distributed AuNPs with an average size of 18 nm (Fig. 2a). On addition of Mn^2+^, however, the particle size increased significantly (average size of 97 nm) as demonstrated in Fig. 2b. This corresponds to the red-shifted high intensity LSPR band at around 530 nm (Fig. 1a, blue curve). Therefore, the LSPR band and the TEM images confirmed that Mn^2+^ induces the growth of AuNPs. Reduction of the added gold ions that are attracted to the AuNPs may account for the particle growth.^25^

The X-ray diffraction (XRD) patterns of the AuNPs were investigated. A 1 cm × 1 cm cut piece of filter paper was soaked in AuNP colloid and AuNPs–Mn^2+^ mixture to allow the deposition of the particles. UV/visible spectroscopy measurements were done for complete deposition of the particles (data not shown). The peaks observed at 2*θ* = 38.47°, 44.54°, 64.44°, and 77.87° demonstrate the diffraction signals of the (111), (200), (220), and (311) crystalline planes of gold ^24^. The peaks at 2*θ* = 22.97° and 34.48° correspond to the (200) and (004) planes of the cellulose-I crystal structure of the filter paper.^24^ Both for the AuNPs and AuNPs-Mn^2+^, the characteristic peaks for the face-centred cubic crystalline structures are observed (Fig. S3).

### 3.2. Optimization of the sensing system

**Fig. 3:**
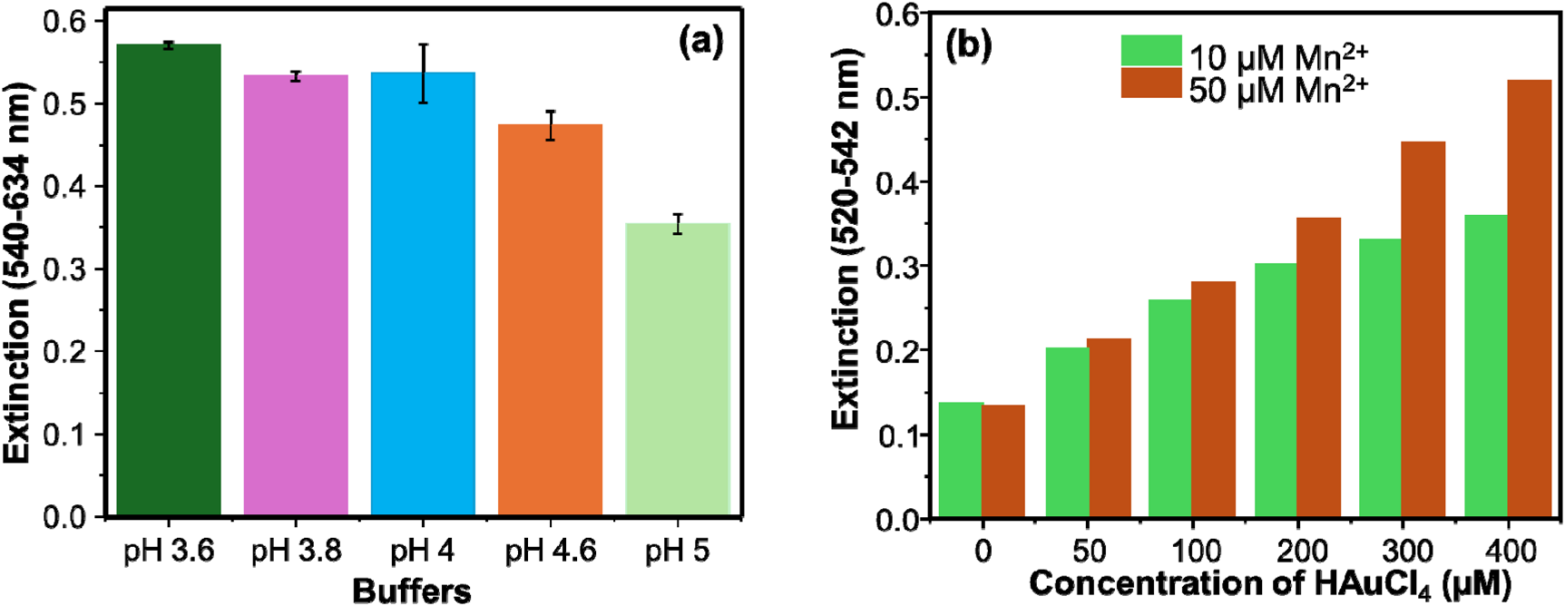
(a) Effect of pH on the intensity of the extinction spectra of AuNPs in the presence of 100 µM Mn^2+^. (b) Effect of HAuCl_4_ on the extinction intensity of AuNPs. Different concentrations of HAuCl_4_ were incubated with AuNPs and Mn^2+^.

Different factors were considered in developing the sensing system. Buffers of different pH ranging from 3.6–5.0 were employed to investigate their effect on the assay of Mn^2+^. Pronounced variation on the extinction spectra of the AuNPs was observed at different buffer pH values. Band shift, broadening of the bandwidth, and change of the plasmonic intensity are observed. The extinction spectra showed a *λ*_max_ difference as big as 93 nm for a pH of 3.6 and 5.0 nm (Fig. S4). The effect of pH on the intensity of the extinction spectra is marginal, as demonstrated in Fig. 3a. These results are ascribed to the variation in the size and shape of the AuNPs. The pH influences the size, polydispersity and morphology of the final particles.^25^ It was found that a pH of 3.6 is the optimal condition for the sensitive detection of Mn^2+^. As the enhancement in the LSPR signal is pronounced in the presence of HAuCl_4_ (Fig. 1a, red curve), the effect of different concentrations of HAuCl_4_ was investigated. The effect was studied systematically for two concentrations of Mn^2+^ (10 and 50 µM) separately. Different concentrations of HAuCl_4_ (0–400 µM) were mixed with AuNPs and Mn^2+^ and were incubated for 30 min. As shown in Fig. 3b, for 0 and 50 µM of HAuCl_4_, there is no difference in the LSPR intensities. A variation in the intensities, however, was observed as the concentration increased, and the highest difference is obtained for 400 µM of HAuCl_4_. In addition to the extinction intensity, bandwidth variations are observed for the same concentration of HAuCl_4_ at different Mn^2+^ concentrations (Fig. S5). Thus, the concentration of 400 µM HAuCl_4_ is used for this study.

### 3.3. Sensitivity and selectivity of the assay

**Fig. 4:**
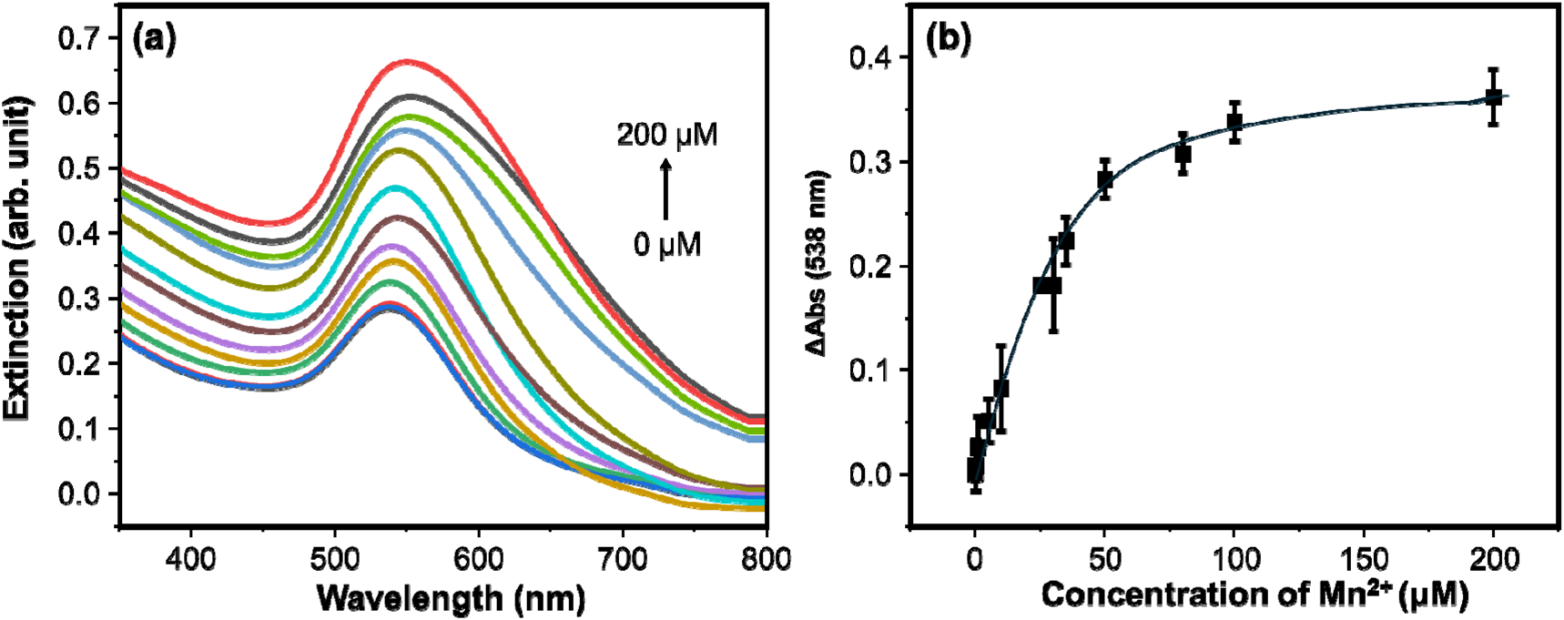
(a) Changes in the extinction spectra of the AuNPs in response to the concentration of Mn^2+^. (b) Concentration profile curve showing the relationship between the extinction spectra difference (*E* − *E*_0_) of the AuNPs and the concentration of Mn^2+^.

The probe mixture of AuNPs and HAuCl_4_ responds to the addition of Mn^2+^. As observed in Fig. 1a, the *λ*_max_ of the colloidal AuNPs redshifted with the addition of HAuCl_4_. The extent of red-shifting (from the colloidal AuNPs) is more pronounced in the presence of Mn^2+^. As shown in Fig. 4a, progressive enhancement in the UV/visible extinction spectra in response to increasing concentration of Mn^2+^ is observed. This shows an increase in the size of the AuNPs, which results in an increment in the extinction coefficient.^22^ Quantitative information for predicting the sensitivity of the AuNP colloids towards variations in the concentration of Mn^2+^ is obtained by plotting the LSPR intensity at Δ*E*_538_ nm (*E* − *E*_0_) against increasing concentration of Mn^2+^ in the range of 0–200 µM. The extinction difference, *E* − *E*_0_, refers to the extinction of the colloidal AuNPs for the blank (*E*_0_) and for the Mn^2+^ (*E*). As shown in Fig. 4b, a good linear relationship between Δ*E*_538_ nm and Mn^2+^ concentration that fits the linear equation *y* = 0.00653[Mn^2+^] + 0.30966 was obtained with *R*^2^ = 0.9999 for the concentration range of 0.5–25 µM. The limit of detection is computed using 3*σ*/*S*, where ? is the standard deviation of the intercept, and S is the slope of the equation, and is found to be 0.295 µM. In addition, a linear relationship was obtained for the concentration range 25–50 µM, and it fits the linear equation *y* = 0.00405[Mn^2+^] + 0.37154 with *R*^2^ = 0.9933.

The LOD and linearity of our method were compared with other reported colorimetric methods that were developed using the LSPR property. As shown in Table 1, our method exhibits comparative results. Importantly, our method avoids modification of the AuNPs and utilizes a non-aggregation-based approach.

**Table 1:** A comparison of the LOD and linear range of our method and previously reported nanomaterials in the determination of Mn^2+^

No	Nanomaterial (colorimetric)	Limit of detection (µM)	Linear range (µM)	Reference
1	AgNPs	0.052	0.1–5.0	29
2	AgNPs	0.020	0.15–15	30
3	SAA-DTC-AgNPs	1.7	5–50	31
4	NTTTA-AgNPs	0.0126	0.05–10	32
5	PVP-AgNPs	0.282	20–80	33
6	AgNPs	0.2	0.1–2.5	34
7	AuNPs	0.295	0.5–25	This work
			25–50	

**Fig. 5:**
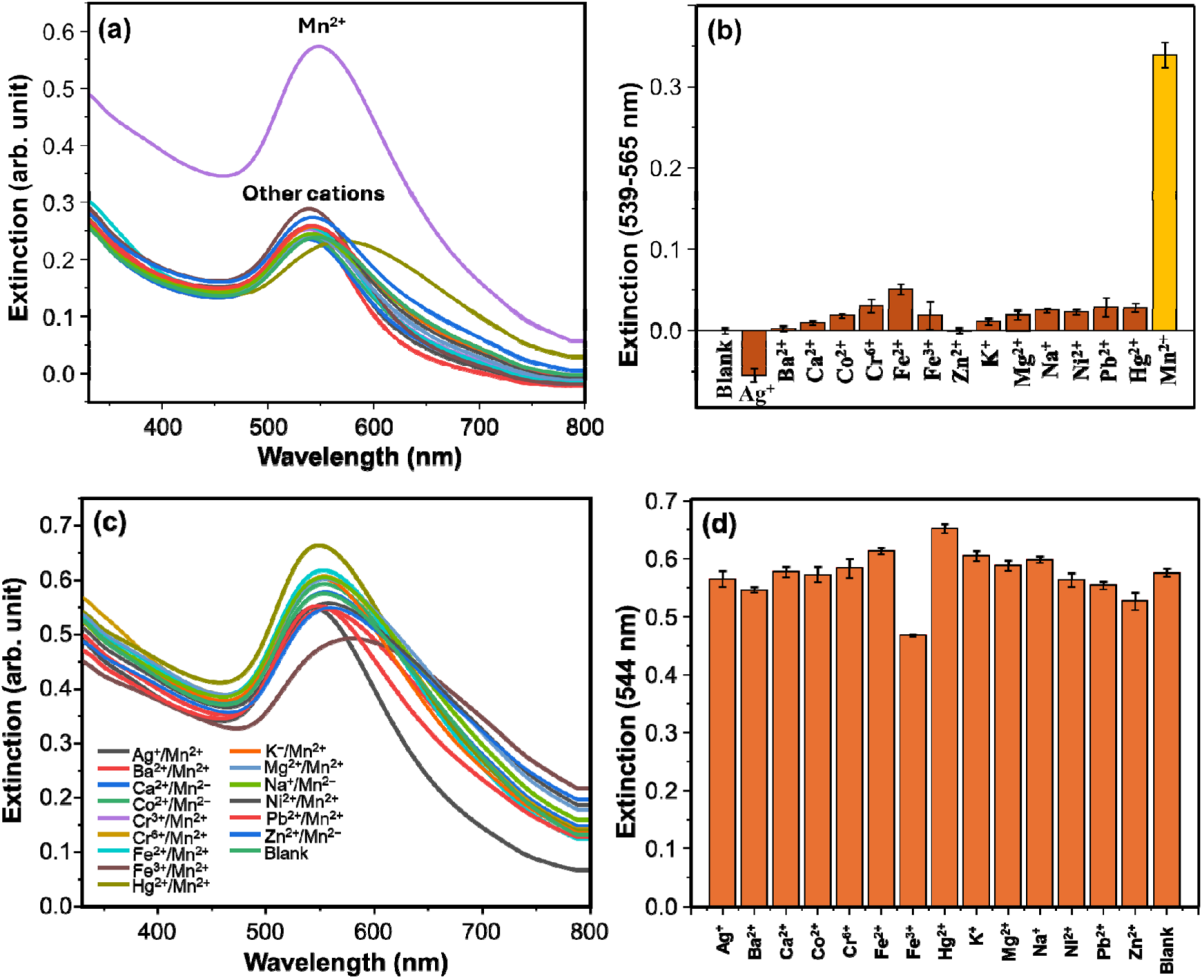
(a) Extinction spectra of AuNPs in response to the addition of various cations in the testing reaction systems. (b) Extinction difference (*E* − *E*_0_) of AuNPs after incubation with different cations, where *E* and *E*_0_ refer to the intensity of the analyte and the blank, respectively. (c) Extinction spectra of AuNPs in response to the addition of various cations and Mn^2+^ (binary system) in the testing reaction systems. (d) Extinction intensity of AuNPs after incubation with different cations in the presence of Mn^2+^.

The selectivity of the method in the detection of Mn^2+^ was studied. The response of the assay towards environmentally relevant metal ions was investigated. Fig. 5a shows the extinction spectra of the AuNPs upon the addition of various metal ions. The presence of the various potentially interfering ions hardly changed the extinction property of the AuNPs, whereas Mn^2+^ significantly enhanced the extinction intensity, showing the selectivity of the assay to Mn^2+^. The LSPR intensity at Δ*E* (539–565 nm) for the tested ions is demonstrated in Fig. 5b. The Δ*E* refers to *E* − *E*_0_, where *E* is the extinction in the presence of metal ions, and *E*_0_ refers to the extinction of the blank. Najeeb *et al.*, (2018) reported that the potential differences in the reduction potential of Mn^2+^ (−1.18 V) and Ag^+^ (0.79 V) can lead to spontaneous reaction of Mn^2+^ and AgNPs.^29^ The gradual increment in the LSPR intensity of AgNPs in the presence of Mn^2+^ strengthens the reduction potential difference observation.^35^ The high reduction potential difference of Mn^2+^ and Au^3+^ favours the reduction of Au^3+^ to Au^0^. Importantly, a recent study demonstrated the ability of Mn^2^^+^ to reduce Hg^2+^ to Hg^0^,^36^ which supports the plausibility of this reduction mechanism. Thus, the selectivity of the assay can be ascribed to the reduction of Au^3+^ to Au^0^ due to the large difference in reduction potentials of Mn^2+^ and Au^3+^. The anti-interference capacity of the assay in detecting Mn^2+^ was investigated using binary ion systems containing Mn^2+^ and another cation. Unlike the single cation testing systems (selectivity test), where the extinction intensities remained comparable with the blank, for the binary systems, the LSPR intensities are comparable with the Mn^2+^ single ion system (Fig. 5c and d). The comparable LSPR intensities for the binary and Mn^2+^ single ion systems indicate that the particle growth effect of Mn^2+^—which is ascribed to the enhanced LSPR intensity—was not hindered by the presence of other cations. This shows high tolerance of the detection system for other tested cations. Therefore, the detection system has excellent selectivity to Mn^2+^ as compared to other tested cations including Ag^+^, Ba^2+^, Ca^2+^, Co^2+^, Cr^6+^, Fe^2+^, Fe^3+^, Hg^2+^, Na^+^, K^+^, Ni^2+^, Pb^2+^, Mg^2+^, Mn^2+^, and Zn^2+^.

### 3.4. Determination of Mn^2+^ in borehole groundwater samples

The applicability of our method for the determination of Mn^2+^ was tested by taking borehole groundwater as a real sample. The borehole groundwater was filtered and diluted ten times with deionized water and used to prepare the desired concentrations of Mn^2+^. UV/visible absorption spectroscopy measurements were done after the spiked Mn^2+^ solutions were mixed with the detection system. The concentrations were predicted by comparing the absorbance with the established calibration curve. The recoveries of the analyses are within the range of 80–110% (Table 2), which is the recommended recovery rate for the studied concentrations.^25^ The method shows high precision as predicted from the low relative standard deviation (RSD), which is below 6.49%.

**Table 2:** Analysis results for the determination of Mn^2+^ in borehole groundwater

Borehole water	Added Mn^2+^ (µM)	Obtained Mn^2+^ (µM)	Recovery (%)	RSD (%)
Sample 1	8.00	8.67	108.4	1.10
Sample 2	12.00	12.14	101.2	6.49

## 4. Conclusion

In this study, a simple, sensitive, and selective method based on a non-aggregated LSPR approach was employed for the determination of Mn^2+^ in aqueous solution and borehole groundwater samples. The extinction property of the AuNPs changes in the presence of Mn^2+^: small AuNPs grow when Mn^2+^ is introduced to the detection system. The method requires no surface modification of the AuNPs, which is a long, tiresome process that is commonly encountered in aggregation-based detection systems of LSPR. This approach opens an avenue for the detection of other metal ions and molecules.

## Conflicts of interest

The authors declare that there are no conflicts of interest.

## Supplementary Material

RA-016-D6RA02740G-s001

## Data Availability

The data supporting this article have been included as part of the supplementary information (SI). Supplementary information is available. See DOI: https://doi.org/10.1039/d6ra02740g.
