# Supplementary material for: Analyte-mediated growth of gold nanoparticles for non-aggregation-based colorimetric detection of manganese(ii)
Source: RSC Adv. 2026 Jun 2;16(33):29932–9. doi: 10.1039/d6ra02740g (PMC13231371; doi:10.1039/d6ra02740g)
Supplement: RA-016-D6RA02740G-s001 [file RA-016-D6RA02740G-s001.pdf]

Supplementary Information (SI) for RSC Advances.
This journal is © The Royal Society of Chemistry 2026

Analyte-mediated Growth of Gold Nanoparticles for Non-aggregation Colorimetric Detection of Manganese (II)

Melisew Tadele Alula*, Mildred Lesang Madingwane

Department of Chemical and Forensic Sciences, School of Pure and Applied Sciences, Botswana International University of Science and Technology, Plot 10071, Private Bag 16, Palapye, Botswana. E-mail: alulam@biust.ac.bw; Fax: +267-4900102; Tel: +267-76126741

Supplementary data

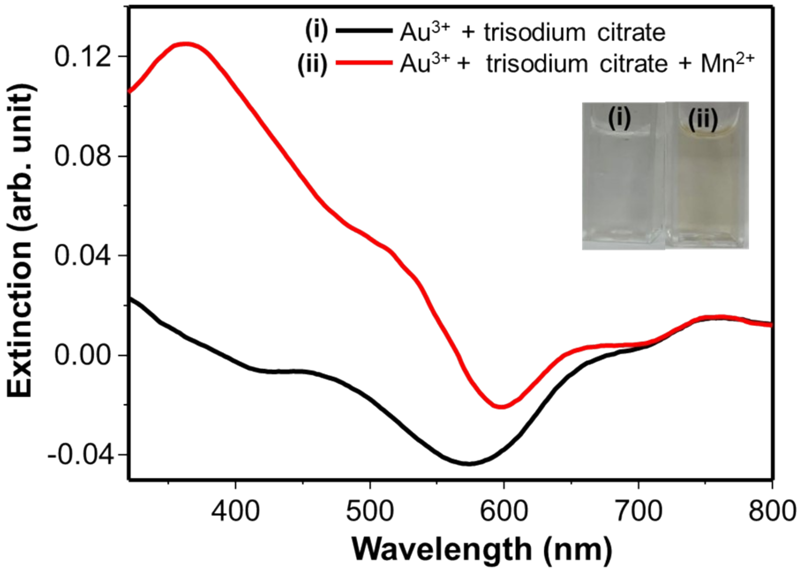

Fig. S1 UV/Visible absorption spectra of $HAuCl_4$ + trisodium citrate and $HAuCl_4$ + trisodium citrate + Mn^{2+} mixtures. The concentrations of $HAuCl_4$, trisodium citrate, and Mn^{2+} are 0.25 mM, 8 mM, and 100 µM respectively. The inset shows the picture of the mixtures.

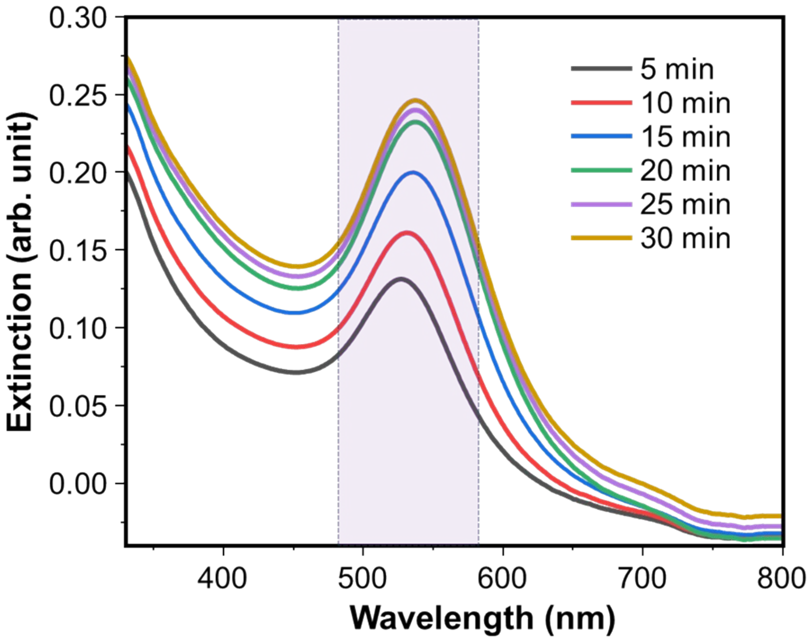

Fig. S2 Extinction spectra of AuNPs evolved with time in the presence of Mn^{2+}.

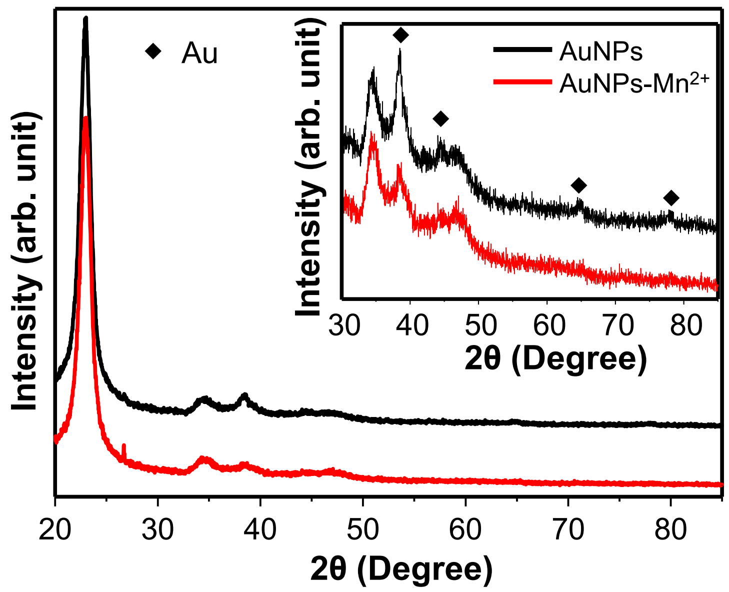

Fig. S3 XRD patterns of AuNPs deposited on filter paper. The inset shows the enlarged XRD patterns.

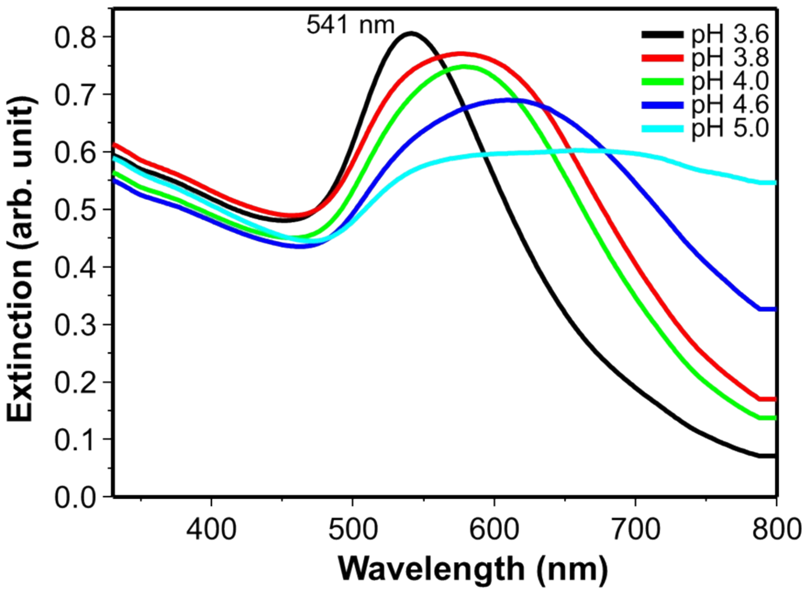

Fig. S4 Extinction spectra of AuNPs after addition of 100 μM Mn^{2+} using buffer of different pH.

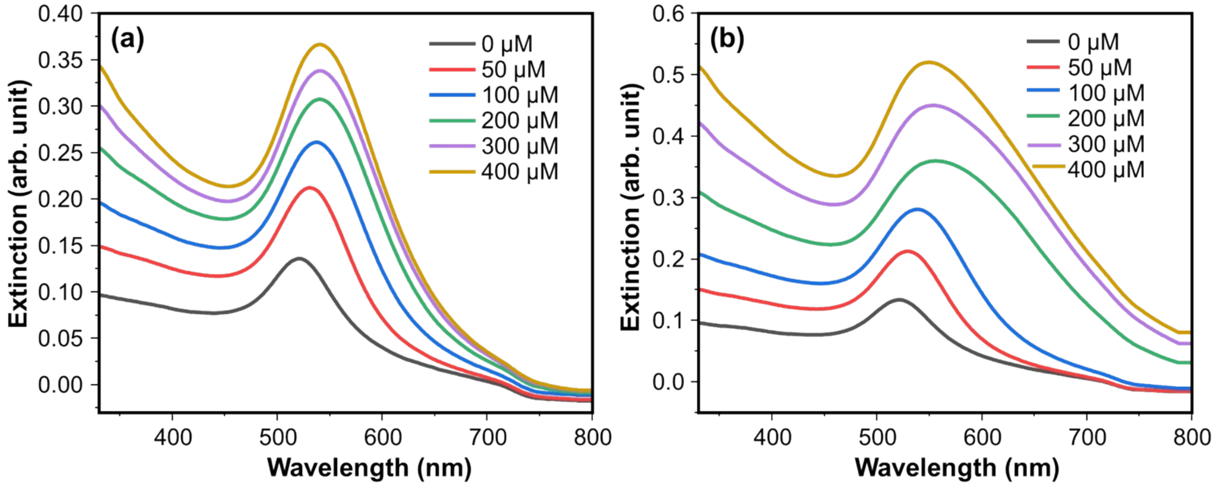

Fig. S5 Extinction spectra of AuNPs showing effect of concentration of $HAuCl_4$ on addition of (a) 10 μM Mn^{2+} and (b) 50 μM Mn^{2+}.
